# Supplementary material for: The implementation of random survival forests in conflict management data: An examination of power sharing and third party mediation in post-conflict countries
Source: PLoS One. 2021 May 3;16(5):e0250963. doi: 10.1371/journal.pone.0250963 (PMC8092654; doi:10.1371/journal.pone.0250963)
Supplement: S2 File — (PDF) [file pone.0250963.s002.pdf]

S2 File. Supplemental discussion of simulation framework and findings

We assessed the performance of the Cox and SRF approaches using several simulated datasets in which the collinearity, number of covariates, and magnitude of the effect of an important binary strata were varied. We examined the number of covariates at 5 and 15, the strata effect magnitude at moderate and high, and the magnitude of collinearity at none and some[1]. Figures S2.1 and S2.2 summarize the results at these lower dimensional scenarios. The strata survival curves for both methods reveal similar information about the differences between strata survival probability. The SRF approach calculates tighter confidence bands than the Cox model, which allows for statistically significant differences to be identified. This is only meaningful in conjunction with a discussion of clinical significance of the difference observed between strata, but note that for all cases considered, there is a moderate or highly influential stratum that was predetermined to be clinically significant. In most cases, the SRF improved the detection of this difference, especially when the true difference was smaller.

---

[1] We constructed simulated data for these scenarios was with the censoring rate at 20%, meaning that 1 of every 5 experimental units can be expected to either last until the end of study or drop-out during the study. We induced collinearity by constructing a covariate as a linear combination of 2 to 3 other covariates added to a normally distributed random error term. We determined the magnitude of collinearity by adjusting the random error and visualizing the relationship between the variables.

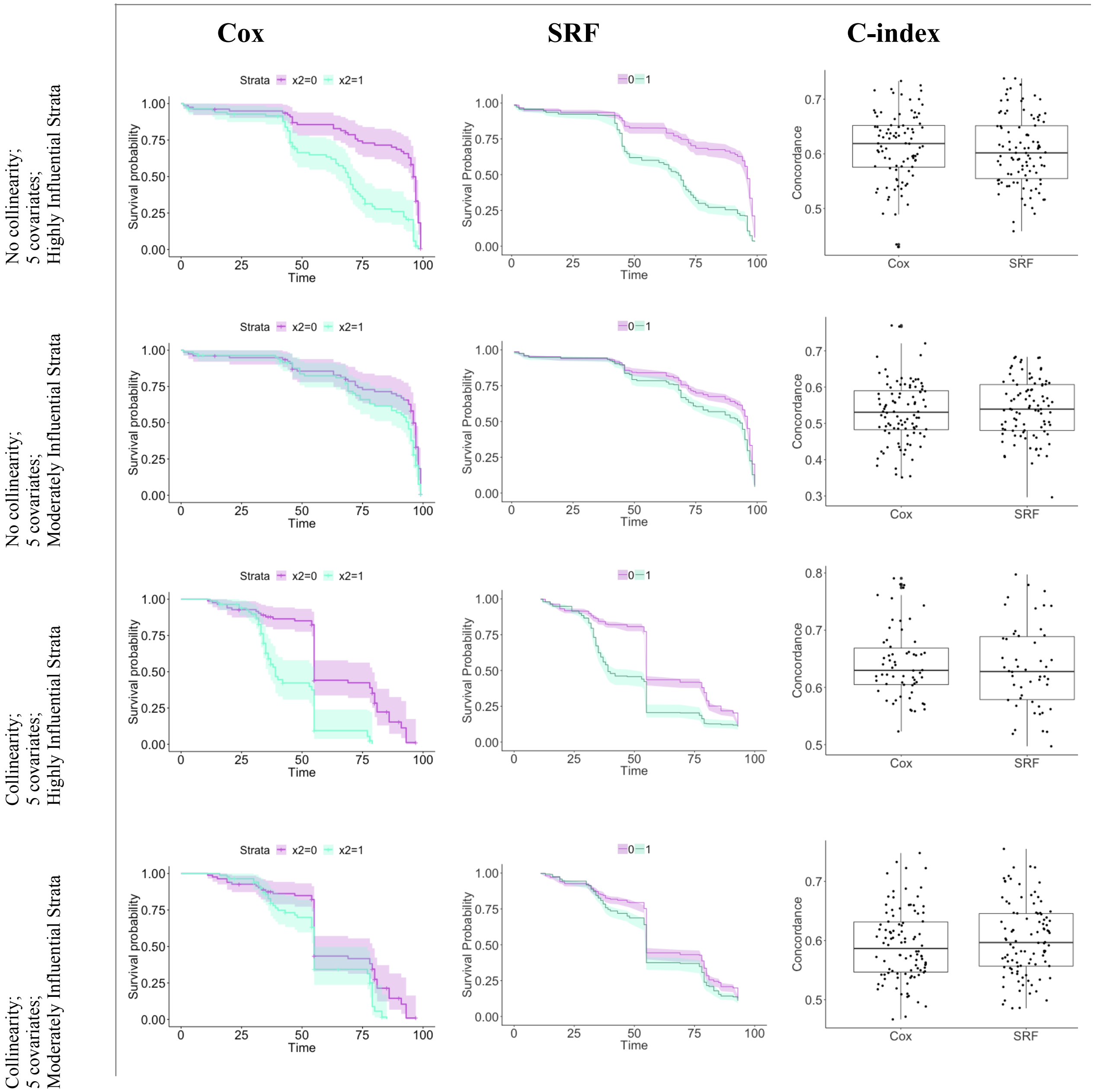

Figure S2.1 Survival curves and concordance assessment of predictive performance for 5 covariate models. Survival curves differ in shape in cases where collinearity or number of variables is altered, since the nature of the simulated data was inherently changed.

Figures S2.1 and S2.2 also provide a comparison of concordance in the right-most column by examining the variance of the concordance indices. We constructed boxplots of concordance indices for each approach using 100 bootstrap-sampled models and then calculating the average

time dependent concordance index of the OOB data on each model. Each dot projected onto the boxplots represents one bootstrap sampled assessment of concordance.

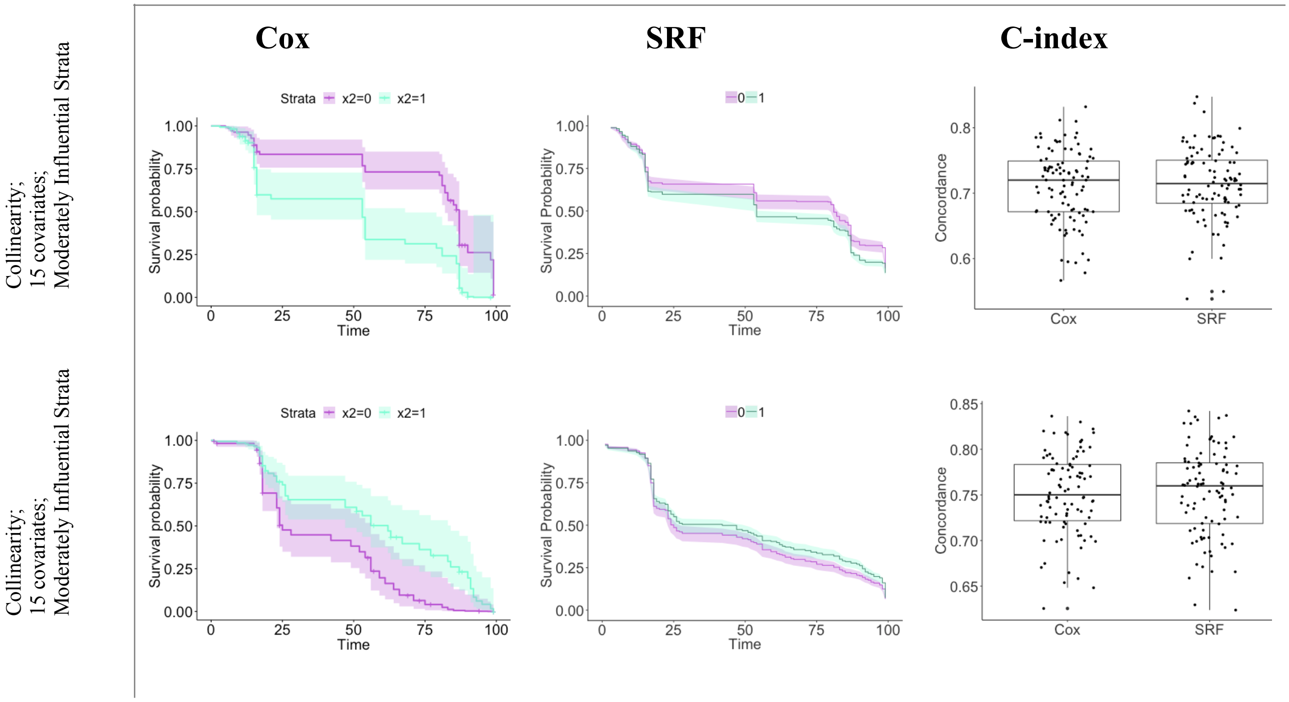

Figure S2.2: Survival curves and concordance assessment of predictive performance for 15 covariate models.

Final Simulation

To complete the comparison of the Cox approach to the SRF, we provide a final scenario highlighting the benefits of the SRF approach. It is well understood that the loss of model interpretability can occur even at a small number of covariates, and this problem exacerbates as the dimensionality increases.

Our most instructive simulation, as presented in the manuscript, incorporates 50 predictors (49 random normal and 1 categorical) on a sample size of 150 observational units with arbitrarily induced collinearity. We simulate a time-to-event (or duration) response variable based on these predictors, where the duration variable is simply the linear combination of the predictor variable values multiplied by their respective effect sizes. To avoid complicating the assessment, we limit the magnitude of most predictor variables' effects to negligibly low effect sizes, and as such, we focus on detecting the predictive effects of only a few continuous variables and primarily the one categorical variable, all of which are determined by construction to be strongly related to duration. Inter-dependencies between predictors are induced by defining a 49x49 covariance matrix with entries of varying magnitudes, the purpose here being to induce collinearity rather than measure its magnitude. The response variable range is defined from t=[1,100]. Observational units that did not experience the event by the end of study are right censored. The censoring rate randomly imposed on the simulated data was set at 0.20.

Figure S2.3 shows a clear binary covariate effect in the SRF survival curves. In the comparison of the 50 variable Cox and SRF models in Figure 4.2, it is observed that the SRF model performs comparably to the Cox approach. What is jeopardized in the Cox approach that is not lost in the

SRF approach is the interpretability of the baseline model. In all trials at this dimensionality, the Cox model had parameter estimate convergence issues that also raise concern with model interpretability. The Cox approach in most applications will require further the use of variable selection and/or regularization to provide meaningful results.

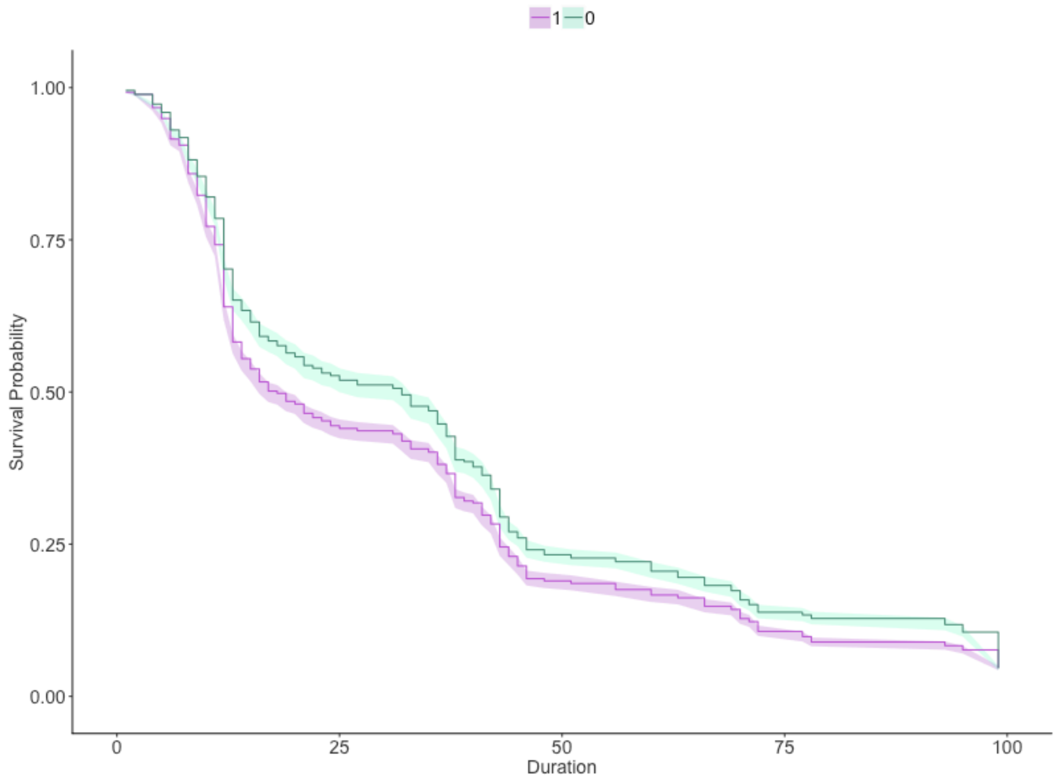

Figure 4.1: Survival curves for the 50 covariate SRF model.

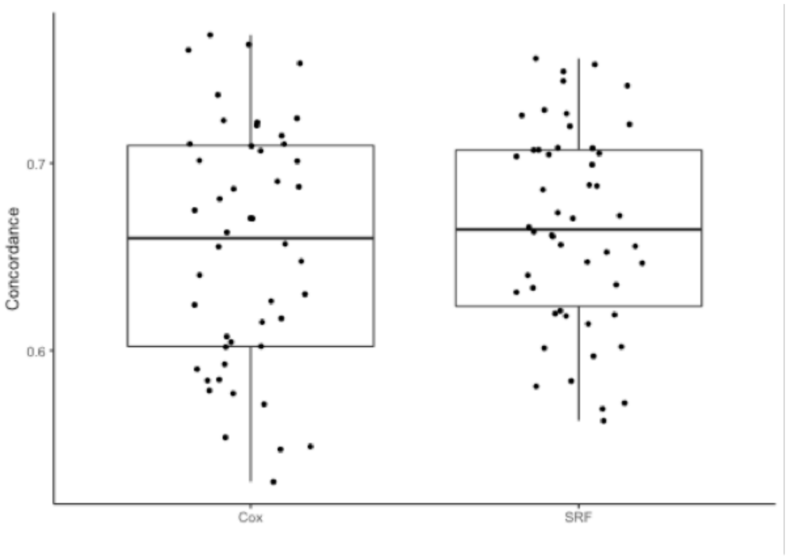

Figure 4.2: Concordance performance assessment. Fifty bootstrap samples of the data were constructed and a Cox and SRF model was constructed on the in-bag data. The out-of-bag data was used to construct concordance predictions.
