# Supplementary material for: The implementation of random survival forests in conflict management data: An examination of power sharing and third party mediation in post-conflict countries
Source: PLoS One. 2021 May 3;16(5):e0250963. doi: 10.1371/journal.pone.0250963 (PMC8092654; doi:10.1371/journal.pone.0250963)
Supplement: Attachment — Submitted filename: Response to Reviewers.pdf [file pone.0250963.s003.pdf]

Reviewer #1: In ``Conflict management data analysis using survival random forests'', Whetten et al. illustrate how to use Random Survival Forests (RSFs) to relax the parametric portion of Cox proportional hazards models in the context of an important question for political scientists and international relations scholars --- namely, the determinants of peace accord durability. While the paper is a welcome contribution to a body of work that illustrates the potential uses of various machine learning (ML) approaches in the study of domain-specific questions, the arguments advanced in favor of RSFs fall short on a few serious respects.

We thank the reviewer for their careful consideration of our work, including their observation that this is a welcome contribution to the literature. We also appreciate the opportunity to respond to this review, and believe this has given the paper greater clarity and value.

Perhaps the most serious concern raised by the manuscript is the authors' emphasis on the issue of predictor correlation and the use of variable importance measures to justify the use of RSFs. There is a well established literature demonstrating how various variable importance measures can fail to correctly identify relevant predictors in the presence of strong correlation among features (see, for instance, Gregorutti et al. 2017; or Strobl et al. 2008). In the context that Whetten et al. study, the power-sharing variables are likely to be correlated (although they show no evidence of this being the case). The authors make no mention of this issue, and they therefore run the risk of leading applied researchers in Political Science astray by this omission.

We agree that this is an important issue to address, and that the audience for this paper would be well served by being more aware of it. Accordingly, we have added a brief discussion (lines 144-150) and included references to both the Gregorruti and Strobl papers.

A second major issue is the lack of discussion of parameter tuning and the risk of overfitting in the context of RSFs. Like other RF approaches, RSFs require selection of tuning arguments, including the number of bagged samples, the proportion of features considered at each splitting node, and the tree-depth. On very large datasets, selecting the most appropriate values for these tuning arguments can be very computationally intensive. Moreover, care must be taken to respect the dependecy structures observed in data (such as those observed in the PSED data, which records multiple observations for the same government) when selecting parameters based on commonly used strategies, such as k-fold cross-validation. These are not ``minimal tuning'' tasks, and they deserve attention.

We agree that tuning is important, and now reference this issue and briefly demonstrate how to approach it in lines 199-205.

We also agree that it is important to pay attention to potential dependence structures, so we have also incorporated an additional explanatory variable, peace agreement location. All peace agreements that occurred in a location with no replications were

marked as “Other” locations. This simplification of this predictor is done to reduce the number of levels of this predictor while still retaining the vital dependency structure mentioned by the reviewer. This choice also allows us to make comparison between countries that observed one conflict versus countries that experienced multiple. This discussion has been added in lines 105-112.

A couple of more minor issues compound these concerns. First, The authors state both that there is ``evidence of improved predictive performance'' of RSFs vs. the Cox model (p. 4, l. 138) and that ``the RSF and elastic net Cox model did not have exceptional differences in computational or predictive performance''. While these statements refer to the non-sparse and the sparse Cox models, respectively, it is an unnecessarily confusing set of statements, and makes me wonder what the measures of predictive performance used were (since the authors leave these unstated).

In lines 170-174, we now clarify that we used bootstrap sampled concordance with B=100 bootstrap samples. The distributions of the concordance measures were compared in our assessment.

Second, the authors never present the true data generating process behind their simulation (which is also unnecessarily vague in terms of the strength of induced correlations), which in turn makes it impossible to discern how well the variable importance measures capture the right set of predictive features in the simulation study.

We believe a balance must be struck here. While we can be transparent to allow a full consideration of the details of our simulation, those details are not critical to an understanding of the survival random forest. Accordingly, we have added a brief statement regarding these details, with a reference to where those details are more fully presented for interested readers in lines 117-119.

Third, there is no discussion of how confidence intervals are constructed for the RSF-based partial dependence plots; I just hope they are based on the infinitesimal jackknife (Wager et al. 2014), which is the most efficient, theoretically-guaranteed approach to building confidence intervals of nominal coverage in RFs.

Delete-d jackknife estimators (Shao and Wu, 1989) were used in the construction of the confidence intervals, as now noted on lines 143-144. This is the recommended and default option available for RSF .

Finally (and this may be a pet-peeve) but there is no such thing as a ``highly significant'' result; under the NHST paradigm, a results is either significant or it is not.

We agree with this preference, and have adjusted it accordingly.

Given these concerns, I am afraid I cannot recommend the current version of the manuscript for publication. However, I encourage Whetten et al. to address these issues, and offer applied researchers in Political Science a more decanted illustration of how ML models can be used to help questions of interest in the discipline.

We have responded to the reviews, and thank the reviewer for helping make this a stronger work.

Reviewer #2: The article provides a useful introduction to RSFs. The work that they present certainly highlight a number of benefits that could come from increases in the application of this approach. The sensitivity of canonical survival analyses is well understood in the literature even if no ideal solution has been agreed on. The alternative that the authors pose here certainly warrants increased attention in the literature.

We thank the reviewer for their careful consideration of our work, and agree that this work will bring increased attention to a beneficial method in the field.

However, a few questions/notes:

- The manuscript spends a notable amount of time highlighting how easy it is to implement an RSF, but more discussion is needed on how to train the model and avoid overfitting (e.g., number of trees, max features for splitting node, etc). Many packages in R and Python enable users to employ a random grid search over a set of hyperparameters, would that be the author's recommendation as well? This question should not be taken as me asking the authors to defend a certain strategy, but rather to highlight that when using these types of models scholars do need to actually have some sort of strategy.

We agree that tuning is important, and now reference this issue and briefly demonstrate how to approach it in lines 199-205, including the method used to implement this tuning.

- The utility of partial dependence plots in highlighting non-linear effects is certainly an advantage but in the context of high dimensional data, relying on PDPs can also be misleading. The maximum number of variables that we can typically examine via a PDP is two (and with a bit more work three). Say that we want to understand how quickly a intra-state conflict will end given the number of rebel groups and strength of the government's military. For the PDP of one of the features, e.g. # rebel groups, we assume that the other features (strength of government's military) is not correlated with the number of rebel groups, which in intrastate conflict research is a false assumption. For the computation of the PDP with a certain number of rebel groups, we average over the marginal distribution which might include a number of rebel groups that is unrealistic for a military of certain strength. Basically, meaning that when the features are correlated, we create new data points in areas of the feature distribution where the actual probability is very low. There are palliatives to this issue in the RF literature, but

again what I think is most important is that readers of this work dont walk away thinking that RSFs are a panacea.

We acknowledge that PDPs are best used to only examine 1-2 features. We have acknowledged this and other drawbacks of this approach in our manuscript, on lines 142-148.

Reviewer #3: The core idea of this paper is to use a random forest variant of survival models as applied to a longstanding question in political science about the duration of accords.

We thank the reviewer for their careful consideration of our work, and believe their feedback has given the paper greater clarity and value.

There are a couple of things that really need to be improved here. First, the figures etc. appear to just be sort of default outputs from R with uninformative labels that are difficult to interpret.

We have made cosmetic upgrades to the multiple figures in the manuscript, primarily renaming variables more clearly in the document.

Second, it is unclear what is new here at all. Neither partial dependence plots nor variable importance are novel to political science or other literatures. The papers the authors cite cover this same ground. If the aim is to make a methods contribution, it's hard to see what is being offered. To the extent there is a methods point being offered, it is that Random forests can be interpreted. But I don't see how that is true. There is no sense in wich these variable importance plots or patial dependence plots can be interpreted in a statistical sense? (Relatedly, where do those confidence intervals in the partial dependence plots even come from? What are their properties?) Remember that random forests are a greedy algorithm, not a true statistical model in a traditional sense. RF are a "black box" in the sense that the outputs have no clear statistically motivated meaning. If I am wrong here, then this should be spelled out in much greater detail and that would be a contribution.

The updated abstract clarifies that the focus here is to introduce this RSF method to a potential new audience in political science research. We have also added a brief explanation about the confidence intervals in partial dependence plots, specifically lines 141-142 for how they are constructed. We agree that RF is not traditional statistics, and notions of "statistical significance" don't apply. However, RFs are not just unexplainable "black boxes" -- see the added lines 60-62 and the added reference to Molnar 2019 for additional discussions. Variable importance plots and PDPs can be understood intuitively even without a formal formula given (and in this way they can maybe be considered even more interpretable than a linear regression coefficient). This is how we have approached them here.

To put a finer point on this, in the application the authors look at how the partial dependence plots find something significant while the Cox model does not. But the sample sizes for those groups are incredibly small, so we probably *want* huge confidence intervals. Perhaps the authors could show that RF has better coverage rates or better power/size visa simulation? But otherwise it seems like it is just overfitting.

Delete-d jackknife estimators (Shao and Wu, 1989) were used in the construction of the confidence intervals. This is the recommended and default option available for RSF models. Several simulations were performed in light of this question early in our project (see Whetten 2018). We identified that SRF models yielded tighter confidence bands than Cox Models. In our simulation the validity of the tighter confidence bands was assessed by artificially varying the magnitude of the effect of the variable of interest in the generation of the simulated data. We found that for a “moderate” effect sizes, the RSF provided stronger conclusions that a significant difference in survival probability was present. We acknowledge this as a disclaimer rather than an advantage of the RSF approach.

Finally, much greater attention is needed to discussing tuning parameter selection.

We agree that tuning is important, and now reference this issue and briefly demonstrate how to approach it in lines 199-205, including the method used to implement this tuning.
