# Supplementary material for: The implementation of random survival forests in conflict management data: An examination of power sharing and third party mediation in post-conflict countries
Source: PLoS One. 2021 May 3;16(5):e0250963. doi: 10.1371/journal.pone.0250963 (PMC8092654; doi:10.1371/journal.pone.0250963)
Supplement: Attachment — Submitted filename: Response to Reviewers.pdf [file pone.0250963.s004.pdf]

1/22/21 Response to reviews of manuscript "The implementation of the random survival forests in conflict management data: An examination of power sharing and third party mediation in post-conflict countries"

We thank the PLOS ONE Editors and reviewers for their time in considering this manuscript. After successfully resolving concerns of three previous reviewers, here we respond to feedback from a Reviewer #4. Our responses are in text boxes, and corresponding edits to the manuscript text appear in pink highlights of the manuscript PDF document. We specifically thank Reviewer #4 for their perspective and believe the resulting revisions have strengthened the manuscript, which we look forward to soon sharing with the readership of PLOS ONE.

Reviewer #4: General:

The paper provide a very brief tutorial and comparison of Random Survival Forests (RSF) models with the Cox proportional hazards model, including a so-called elastic net version of the Cox model. The comparison uses simulated data and a case study using the Power-Sharing Event Dataset (PSED). The authors claim that the RFS is the best "off-the-shelf" method for computational political scientists (under less optimal conditions), and that there is a added interpretative benefits compared to (e.g.) the elastic net version of the Cox model. In addition, they also claim to find significantly higher survival probabilities for certain peace agreements (for the PSED).

As a tutorial, the discussion is a bit short, and to get a complete introduction it is required to look up some of the references. This is not necessarily a problem, and the paper work well as a quick introduction to some of the main themes related to RFS. However, based on the discussion in the paper, some of the claims made about the RFS appear a bit too strong and unjustified. Also, the simulation study, and the corresponding discussion, is a bit short and perhaps too one-sided to provide a complete discussion. I understand that the main part of the simulation study is presented elsewhere, but it would have been nice with a more thorough discussion and perhaps also a more extensive literature overview to make this (as a tutorial) more complete and self-contained.

We have aimed for a balance here, focusing our discussion more on general principles, which we believe will be more useful for the intended audience rather than technical details. We have included additional discussion of the simulation and literature review, and hope the reviewer can appreciate the intended balance.

In addition, I think there are issues related to the case study that are not well discussed or explained in the paper:

1) Problems and consequences related to multiple observations/agreements within the same country.

2) And the introduction and use of the location variables and especially the "Other" variable in the model.

3) The surprisingly tight confidence intervals around the survival functions for the RFS.

These issues are discussed more below.

We have endeavored to respond to each of the concerns below, and thank the reviewer for their perspective.

Abstract:

Is the ranking of peace agreement criteria importance really that novel? Is this because it has not been commonly used in this community before? Also, is the ranking very different from what would be the induced ranking from the elastic net Cox regression coefficient trace plots?

The novelty is in the use of RSF for ranking peace agreement criteria, not in the act of ranking. This clarification has been made in the abstract.

We thank the reviewer for highlighting their concern here, and a new paragraph, discussing the ranking of variables in the RSF procedure was added to the discussion section following the 3rd paragraph in an attempt to address this concern. In short, the primary purpose of trace plots to reflect stability of coefficient estimates, rather than assess the predictive importance of variables on the response. See lines 327-339

Ranking predictor variables is not as clear in a traditional regression-type model as it is in a Random Forest-type model. Is a larger effect size with a smaller standard error better than a moderate effect size with larger standard of error? Is it enough to sort on p-value? (This approach would fail in the presence of high collinearity among important predictors.) Does a larger effect size guarantee greater improvements to classification and MSE performance than a smaller effect size? (In our experience this is not the case as sometimes a variable that has lower significance and/or smaller effect size may still yield highly important improvements to performance in an RF model where the RF models finds that this variables improves predictions for some minority of observational units) In addition, we have seen examples of the subjectivity of the choice of variables in regularization models (like lasso and elastic net). While we do not believe this is worth emphasizing in the manuscript (where we instead refer simply to subjectivity concerns around line 334), for the purpose of addressing the reviewer concern, we can relate a generic example -- if two important predictors are collinear, their estimated coefficients can of course be unreliable, and it can happen that the lasso/elastic net model removes the less stable one and hence eliminates the strong collinearity between these two variables that was yielding alarming.

It is a bit unclear what is viewed as new regarding the justification for the "robust interpretability and competitive performance" for the RFS in this paper.

The novelty is in the application of RSF for such data in the field of political science. Our findings demonstrate a scenario exhibiting the interpretability and performance of RSF for such data. Our updated Abstract puts this novelty in better context.

Introduction:

In my opinion, the discussion of the RFS is a little too sensational based on what is presented (as evidence) in this paper. This could be be solved by a more complete discussion and litteratur review; I assume there are several RFS studies/papers outside political science that could be of interest here.

> We agree it is important to avoid over-sensationalizing any given method, including RSF. We have added additional mention of limitations / challenges in the use of the RSF. See lines 33-36 and 163-167.

Materials and methods:

* Random survival forests

* The Cox model

* The Power-Sharing Event Dataset:

The introduction and construction of the location and "Other" variables to correct for potentially dependent peace agreements appear suboptimal. It is also unclear how this is actually included in the model. In addition, the motivation for this correction and justification arguing that this will solve the problem is unclear?

> We thank the reviewer for alerting us to the needed details about the use and construction of this variable. We have made adjustments in the corresponding paragraph added at the end of the "The Power-Sharing Event Dataset" subsection. See lines 118-136.

Would a multi-state model be more suitable here?

> This is a fundamentally different research question, and one that we agree may be interesting to consider in a future work. We have mentioned this possible future research question in the paragraph added at the end of the "The Power-Sharing Event Dataset" subsection. See lines 121-124

Also, by introducing the variable "Other" in the model, are you not potentially including (part of) the response variable into the covariates? I understand that this does not have to be the case, but could this variable indirectly contain information about the (future) survival? Moreover, from the perspective of doing predictions (for e.g. a new case/country), this, that there will only be one peace agreement (if I understood the definition correctly), is not something that is known in advanced.

"Other" is just one possible level of the location variable, representing countries with only one peace agreement. The fact that these countries could tend to have systematically higher or lower response variable values is precisely the reason to include these countries in the same level of the location variable. We agree that for the purposes of prediction, for new peace agreements in a given country it would not necessarily be known if other peace agreements for the country would be forthcoming. However, accounting for dependence among observations (by the inclusion of the location variable) does allow for a clearer understanding of the predictive effects of the other predictor variables in the model, and that is the key.

We have made further adjustments to this section to bring light to this topic. See lines 125-131

To avoid such problems and/or other problems related to dependency between observations for the same country, would it make more sense to study "time to first recurrence of violence" on the reduced dataset of 41 countries?

That would involve a different research question, and given the additional data for subsequent peace agreements in countries whose first post-1988 peace agreements failed, it would not account for the available data. Our efforts here are to provide an effective analysis that comprehensively analyzes peace agreements from this era. See lines 131-136

Results:

* Simulations and RSF Interpretation

Is it correct that the main finding in the simulation study is that "standard" estimation of the Cox model fail to converge when there is high level of multicollinearity? This is not very surprising, there are solutions to such problem, and, by itself, this is not a very convincing argument (although impractical) against using the Cox model. Are there other main insights from the simulation study that should also be included here?

The key is that RSF methods are excellent off the shelf methods and the challenge is to effectively visualize the off the self findings in such a way that collinearity is accounted for. We acknowledge that although we avoid the need to assess model diagnostics in this approach, there is still some challenging work to appropriately visualize the finding. We have made additional and clear mention of this topic. See lines 33-36 and 163-167

We have also added a supplemental file S2 that presents more discussion of the simulation framework and findings. We consider this supplemental because it is not central to the main focus of this paper. Even so, we do not dwell on technical details in the S2 File. We hope the reviewer can appreciate the balance to keep the manuscript's focus.

In relation to Fig. 2, how does the induced ranking of the coefficient from the coefficient trace plot for the elastic net selected Cox model compare to the variable importance ranking? Is the ranking the same?

Trace plots are best suited to show the stability of the estimated coefficient rather than any notion of predictive performance. More information on this topic as been added to the manuscript based on previous feedback from the reviewer (see lines 327-339). It is possible to try to define some notion of variable importance from the elastic results, but this prone to subjectivity, and wouldn't necessarily be appropriate to compare directly to the traditional definition of variable importance used in RF methods. The RSF approach eliminates this subjectivity that would need to be used in the elastic net cox model.

If the partial dependency plot in Fig 3. is based on the simulated data, how does the results correspond to the model used in the simulations? Is the conclusions/observations in accordance with how the simulation experiment was designed? This is not clear from the text.

We thank the reviewer for bringing attention to this. Lines 178-179 now mention this, without focusing on technical details.

* PSED Analysis

Is it possible to quantify this statement:

"The RSF model shows evidence of improved predictive performance over the baseline Cox model, which comprised of all variables in the original PSED."

I see that concordance is alluded to below, but there are no numeric values(?) to indicate if the difference is of any practical importance. Why not use additional measures, e.g. Brier score?

We have added a brief summary of the performance box plots (using the median, Q1 and Q3 concordance.

The confidence bands shown in Fig. 6 are surprisingly tight, especially when compared to Fig. 4 and what I obtain by a simple and quick re-analysis using the same data and standard models. In general, tighter confidence intervals is not evidence that a model is superior, these intervals could simply be wrong and/or have incorrect coverage probabilities (and interpretation); and in that sense be useless. Therefore, the following statement is questionable:

"The RSF model in many cases improves our ability to identify true differences between strata if they exist, especially with sparse strata."

And I do not see that there is any reference or discussion justifying that this is a valid and general statement.

Moreover, if this is actually the case, and the confidence bands have the correct interpretation, this is so surprising that it would be of independent interest to understand this in more details. For example, why is the RFS able to use the information so much more efficiently and/or what is wrong with the (assumptions) underlying the Cox model?

We greatly appreciate the reviewer catching our miscommunication in our argument here. A major modification to this section has been made to clarify the findings here clarifying the strengths and weaknesses of our model/method. See lines 244-257.

The conclusion at the end of page 7 feels a bit strong. I assume that this is not a truly randomized experiment. A more complete discussion regarding the validity of the conclusions focusing on the quality of data and assumptions underlying the analysis would be appreciated.

We believe the reviewer is referring to the discussion about the interaction between peace agreement location and conflict duration, and we have tempered the conclusions there, with a comment about the observational nature of these data. See lines 298-304

Discussion:

Is it possible to use the trace plots for the elastic net selected Cox model to interpret the importance of covariates? And, how and in what sense is the RFS providing an important interpretative advantage?

We believe that our response to the earlier comment about the trace plots should apply here as well (see lines 327-339). As to the RSF providing an important interpretative advantage, we have added a brief example in the Discussion section regarding the RSF's ability to identify potential interaction effects, which leads to additional discussion in the remaining paragraph there. See line 340.

What is the practical importance of the "durationDY" variable? And, could the interpretation of this and other covariates be affect by having multiple observations from the same country in the same analysis?

We have acknowledged other ways in which interdependence of peace agreements can be implemented, and we have provided further clarification on the practical importance of dyadic conflict duration. See lines 349-360.

We thank the reviewer for their thoughtful and thorough remarks, and we feel that it has improved the quality of our work.
