# Supplementary material for: The implementation of random survival forests in conflict management data: An examination of power sharing and third party mediation in post-conflict countries
Source: PLoS One. 2021 May 3;16(5):e0250963. doi: 10.1371/journal.pone.0250963 (PMC8092654; doi:10.1371/journal.pone.0250963)
Supplement: Attachment — Submitted filename: Response to Reviewers.pdf [file pone.0250963.s005.pdf]

PLOS ONE manuscript “The implementation of the random survival forests in conflict management data: An examination of power sharing and third party mediation in post-conflict countries”

Response to reviewers

04/03/21

We thank the reviewers for their time and expertise in considering this manuscript. The text boxes below include our responses to specific reviewer comments, and we look forward to bringing this work to the PLOS ONE readership.

<u>Reviewer #5:</u> I like the idea of introducing new methodologies in fields that are not fully aware of the latest available data analysis tools. This is why this article is interesting and pertinent even though the data analysis example has limitations.

We thank the reviewer for their time and expertise in considering this manuscript. We share the interest in introducing statistical methodologies (in this case, random survival forests) to fields in which those statistical methods have not previously seen widespread usage (in this case, political science).

The multiple peace agreements in some countries potentially induce dependence among the observations. Adding a dummy variable for the country is reasonable. Our However, any inference (i.e. test of hypothesis, confidence intervals) are questionable because of that. The authors should clearly mention this. Methods for clustered data exist but they would be out of the scope of this paper. The authors should at least mention some of them. Frailty models are well-known and can be used with (semi-) parametric models like Cox. For trees, Fan et al. (2006) propose a way to handle such data; see also the R package MST for an implementation.

We agree and have added these references to the text. We have listed Fan (2006) and Calhoun (2018) for multivariate survival trees, and we have added the additional disclaimer in lines 137-143.

The right way to evaluate the predictive performance and compare the models is to use test data that are not used to fit the model. Since the sample size is so low (n=79), using cross-validation might be the only choice. The Brier score and C-index are the two most popular performance methods and should be used.

We have followed the procedure for model performance assessment outlined in Ishwaran 2008 in which we construct a collection of N bootstrap samples of the data, fit each model to the bootstrapped data, and then assess the predictive accuracy on the “out-of-bag” observations. Theory on bootstrap sampling has shown that approximately 1/3 of the data is excluded in each bootstrap sample, and as such this approach holds the same foundational principle of classical cross-validation which requires prediction onto observations that are left. In this approach, the C-index is used to evaluate the predictive accuracy of the model for each bootstrapped sample. This is already stated in lines 211-215. We have added a reference to Ishwaran (2008) in line 216 to emphasize the consistency of this approach with existing literature. We have also added (in lines 216-218) a reference to Chen et al. (2012), which presents several predictive performance measures for survival analysis models, including the Brier Score.

The use of out-of-bag C-indices to validate our models is also explained more thoroughly in the S2 technical supplement.

Reference

Fan, Juanjuan, et al. "Trees for correlated survival data by goodness of split, with applications to tooth prognosis." Journal of the American Statistical Association 101.475 (2006): 959-967.

We appreciate the reference suggestion and have included it in the text.

Reviewer #6: I'm a new reviewer for this manuscript. I've read the responses the authors provided to the reviewer suggestions from earlier rounds. I've also read the manuscript carefully. The presentation appears thorough to me as do the responses to the comments from the other reviewers. I see no major issues to point out, so at this late stage (from my perspective) I will not provide any minor suggestions for the authors of the manuscript. I will provide one very small suggestion that the authors can choose to ignore if they disagree: generally I recommend very detailed figure captions. The authors' captions are pretty thorough but could stand a bit more detail. The table could use a more detailed caption as well. Again, this is a very very minor point. Otherwise, I'm satisfied with what I've seen in terms of the updated manuscript and the response to the earlier reviewers.

We have added an additional sentence to the end of Fig 1, Fig 2, Fig 4, Fig 6 captions.
We have added two sentences to end of Fig 3, and Fig 5, Fig,7 Fig 9 captions.

We agree that the descriptions in many of these captions are slightly lacking, and we hope that the reviewer finds our additions beneficial to the paper.
